# Automated Operative Phase and Step Recognition in Vestibular Schwannoma Surgery: Development and Preclinical Evaluation of a Deep Learning Neural Network (IDEAL Stage 0)

**DOI:** 10.1227/neu.0000000000003466

**Published:** 2025-04-30

**Authors:** Simon C. Williams, Dorothée Duvaux, Adrito Das, Siddharth Sinha, Hugo Layard Horsfall, Jonathan P. Funnell, John G. Hanrahan, Danyal Z. Khan, William Muirhead, Neil Kitchen, Francisco Vasconcelos, Sophia Bano, Danail Stoyanov, Patrick Grover, Hani J. Marcus

**Affiliations:** ‡ Victor Horsley Department of Neurosurgery, National Hospital for Neurology and Neurosurgery, London, UK;; § UCL Hawkes Institute, University College London, London, UK;; ‖ The Francis Crick Institute, London, UK; ¶ Institute of Neurology, Institute of Brain Repair and Rehabilitation, University College London, London, UK

**Keywords:** Surgical workflow, Operative workflow, Machine learning, Neural networks, Artificial intelligence, Computer vision, Vestibular schwannoma, Acoustic neuroma, Retrosigmoid

## Abstract

**BACKGROUND AND OBJECTIVES:**

Machine learning (ML) in surgical video analysis offers promising prospects for training and decision support in surgery. The past decade has seen key advances in ML-based operative workflow analysis, though existing applications mostly feature shorter surgeries (<2 hours) with limited scene changes. The aim of this study was to develop and evaluate a ML model capable of automated operative workflow recognition for retrosigmoid vestibular schwannoma (VS) resection. In doing so, this project furthers previous research by applying workflow prediction platforms to lengthy (median >5 hours duration), data-heavy surgeries, using VS resection as an exemplar.

**METHODS:**

A video dataset of 21 microscopic retrosigmoid VS resections was collected at a single institution over 3 years and underwent workflow annotation according to a previously agreed expert consensus (Approach, Excision, and Closure phases; and Debulking or Dissection steps within the Excision phase). Annotations were used to train a ML model consisting of a convolutional neural network and a recurrent neural network. 5-fold cross-validation was used, and performance metrics (accuracy, precision, recall, F1 score) were assessed for phase and step prediction.

**RESULTS:**

Median operative video time was 5 hours 18 minutes (IQR 3 hours 21 minutes-6 hours 1 minute). The “Tumor Excision” phase accounted for the majority of each case (median 4 hours 23 minutes), whereas “Approach and Exposure” (28 minutes) and “Closure” (17 minutes) comprised shorter phases. The ML model accurately predicted operative phases (accuracy 81%, weighted F1 0.83) and dichotomized steps (accuracy 86%, weighted F1 0.86).

**CONCLUSION:**

This study demonstrates that our ML model can accurately predict the surgical phases and intraphase steps in retrosigmoid VS resection. This demonstrates the successful application of ML in operative workflow recognition on low-volume, lengthy, data-heavy surgical videos. Despite this, there remains room for improvement in individual step classification. Future applications of ML in low-volume high-complexity operations should prioritize collaborative video sharing to overcome barriers to clinical translation.

**ABBREVIATIONS:**
AI: artificial intelligence
CNN: convolutional neural network
LSTM: long–short-term memory
ML: machine learning
RNN: recurrent neural network
VS: vestibular schwannoma.

Historic paradigm shifts in the operative management of vestibular schwannoma (VS), such as the adoption of the operating microscope and use of intraoperative neuromonitoring, have resulted in a significant reduction in morbidity and mortality.^1,2^ Despite these advances, resection of VS remains a high risk and challenging operation, with outcomes remaining static in the past decades.^3^ Furthermore, the low-volume, high complexity nature of VS resection, coupled with the increasing transfer of expertise to centers of excellence, has brought concerns regarding limited exposure and training opportunities.^4,5^

Artificial intelligence (AI) is poised to transform the way that modern surgical care is delivered, and carries with it opportunities in surgical outcomes and training.^6-8^ Of particular note to surgeons is the opportunity for AI platforms to interpret and process operative video—a branch of AI termed computer vision.^8^ Increasingly, operative video is being seen as a vast mine of untapped data.^9^ Many forms of surgery such as endoscopic or microscopic surgery, such as in VS resection, are uniquely placed to benefit from AI video analysis in that the operative field is displayed in its entirety in video format. Operative videos can be labelled and used to train AI models for a range of outputs, including detecting surgical instruments,^10^ anatomic labelling,^11-13^ predicting phases and steps of operations,^14,15^ and recognizing surgical tasks and situations.^15-17^ These advances have been demonstrated for cataract surgery,^16^ pituitary surgery,^14^ and extensively in laparoscopic general surgery.^6,16,17^ The process of deconstructing operations into discrete phases and steps is known as “operative workflow analysis” and is amenable to AI assistance.^18^ The benefits and proposed applications of AI-assisted operative workflow analysis are wide reaching, including AI-assisted evaluation of surgical performance, training and education, and intraoperative guidance.^18-22^

AI-assisted workflow analysis may improve surgical care and training in VS resection. Previous work established an expert-derived Delphi consensus of the operative phases and steps of the retrosigmoid surgical approach to VS resection.^23^ This study builds on this work, presenting an AI model that uses machine learning (ML) to identify relevant phases and steps of the retrosigmoid approach to VS resection. The low-volume, high complexity nature of VS surgery makes it uniquely placed to benefit from intraoperative guidance, simulation, and training opportunities afforded by automated operative workflow analysis.^24^ The aim of this IDEAL (Idea, Development, Exploration, Assessment, Long-term follow-up) Stage 0^25^ (preclinical) study was to develop a ML model capable of automated operative workflow analysis of operative videos of VS resection through the retrosigmoid approach. In doing so, this would represent the first demonstration of automated workflow analysis in lateral skull-base neurosurgery, while also showcasing ML-based workflow prediction in data-heavy operative videos.

## METHODS

This work is reported in accordance with CLAIM-AI (Checklist for Artificial Intelligence in Medical Imaging)^26^ and IDEAL frameworks.^25,27^ Video annotation was conducted in accordance with the SAGES (Society of Gastrointestinal and Endoscopic Surgeons) consensus framework.^28^

### Study Design and Summary of Methods

A preclinical (IDEAL Stage 0) development and evaluation design was used. Operative videos of retrosigmoid VS resection were prospectively collected at a tertiary-academic neurosurgical center over a 3-year period. Videos then underwent temporal annotation with an agreed set of phases and steps.^23,29^ Annotated videos were used to train a deep learning AI model in a supervised learning methodology. The ability of the model to accurately identify phases and steps was then tested on a subset of unseen operative videos. Metrics for model performance are presented descriptively.

### Data Collection: Operative Videos

Operative microscope videos of retrosigmoid VS resections were prospectively collected over a 3-year period (August 2020-August 2023) at a tertiary-academic neurosurgical center. Inclusion criteria were patients aged 18 years or older who underwent VS resection through a retrosigmoid approach. Exclusion criteria included patients for whom histological analysis later diagnosed a different tumor type, ie, non-VS, translabyrinthine approach, lower-cranial nerve schwannomas, aged younger than 18 years, and incomplete videos (whole phase or more missing). An intended sample size of >20 was set. Written informed consent was obtained from patients before their operative videos being recorded. Approval was granted through our Trust Local Governance Committee in accordance with General Data Protection Regulation. Baseline data including age, sex, and length of operation (minutes) were recorded.

Operations were led by 1 of 5 attending surgeons (2 consultant neurosurgeons and 3 consultant Ear, Nose, and Throat surgeons). Operative videos were recorded in 1920 × 1080 pixels using either a ZEISS Kinevo900 operating microscope (CarlZeiss Co) or a ZEISS OPMI Pentero800 operating microscope (CarlZeiss Co). Intraoperative videos were then exported (MPG and MP4 format) onto an encrypted hard drive and uploaded to TouchSurgery^TM^ (Medtronic), a cloud-based platform for video storage and annotation.

Our team's standard operating procedure for retrosigmoid excision of VS is presented in **Supplemental Digital Content 1** (http://links.lww.com/NEU/E752).

### Ground Truth Labelling: Operative Video Annotation

Operative videos underwent deidentification by the TouchSurgery^TM^ platform. Annotation was conducted in accordance with SAGES consensus framework, and temporal annotation labels were made in-keeping with SAGES consensus definitions (Table 1).^28,30^ In this study, phases and steps were labelled, whereas tasks and actions were not labelled.

**TABLE 1.** Operative Workflow Temporal Annotation Definitions

Event	Definition
Phase (generic)	The highest-level temporal component of an operation comprising a major surgical event, such as “Approach and Exposure,” “Tumor Debulking,” and “Closure”
Step (procedure-specific)	A single or set of linked procedures or actions that are procedure-specific and collectively achieve a surgical objective. For example, “Cisterna magna opening” is a step within the “Approach” phase of a retrosigmoid resection
Task (generic)	A subcomponent of a step, composed of a series of actions to accomplish a step goal. For example, “Retraction of cerebellum,” and “Arachnoid dissection” are tasks required to achieve the step of “Cisterna Magna Opening”
Action (generic)	A primitive component of a task, comprised a series of physical actions required to complete a task, and often linked to a single motion

Derived from SAGES consensus statement on video annotation.^29^

Videos were labelled using 3 phases (Approach and Exposure, Tumor Excision, Closure) derived from an expert Delphi consensus^23^ (**Supplemental Digital Content 2** [http://links.lww.com/NEU/E753]). Within the Tumour Excision phase , dissection and debulking steps were labelled. Idle time, defined as when there is no surgical action within view of the operating microscope, is also reported.^28^ A summary of the phase and step labels used to annotate our dataset is presented in Table 2

**TABLE 2.** Labelled Phases and Steps and Temporal Definitions

Retrosigmoid approach
Phase: Approach and Exposure—labelled from beginning of operative video up until point of first tumor debulking
Phase: Tumor Excision—Labelled from first to the last instance of tumor resection (e.g. with microscissors, CUSA, or dissecting instruments)
○ Step: dissection (inferior)—labelled during microsurgical dissection of the inferior anatomic pole of the tumor
○ Step: dissection (superior)—labelled during microsurgical dissection of the superior anatomic pole of the tumor
○ Step: dissection (medial)—labelled during microsurgical dissection of the medial anatomic pole of the tumor
○ Step: dissection (lateral)—labelled during microsurgical dissection of the lateral anatomic pole of the tumor
○ Step: tumor debulking—labelled during physical tumor removal (e.g. with microscissors, CUSA, or dissecting instruments)
Phase: closure—labelled from last instance of tumor debulking and excision onward (excluding idle time)
Idle time—labelled when there is no action visualized within the view from the camera (e.g. prolonged blurry image, microscope pointed away from patient)

CUSA, Cavitron Ultrasonic Surgical Aspirator.

Ground truth (reference standard) labelling of training and test video datasets was independently performed using TouchSurgery^TM^ by 2 authors (SCW, SS). The beginning and end of each phase and step were manually timestamped, resulting in temporal segmentation. Annotations were subsequently reviewed by 2 skull-base neurosurgeons (PG, WM), and any disagreements resolved through discussion. No inter-rater variability calculations were performed because the above methodology resulted in a single consensus-derived annotation.

### Model Development and Evaluation

ML models were created for 3 tasks: (1) recognition of the 3 phases, (2) recognition of the broad 2 steps of dissection and tumor debulking, and (3) recognition of the intratumor debulking 5 steps as described in Table 2. We aimed to train our ML models using only temporally annotated data (and not other labels such as anatomy or instrument labelling) to focus on task-specific outputs, enabling a clear and direct relationship between the input data (surgical video segments) and the predicted outcomes (operative phases and steps), simplifying the model and improving interpretability.

The 21 videos were converted into a series of images with a sample rate of 1 fps (frames per second) and a resolution of 224 × 224 pixels, as a compromise between dataset size and computational time. For task (1), 3-phase recognition, images were sampled at 1 frame per 25 seconds to allow for improved temporal feature extraction in temporal based models. Operations were mirrored horizontally to standardize orientation of anatomy across all procedures. 5-fold cross-validation was used to ensure model consistency, with a 17-videos training to 4-videos testing split for 4-folds and a 16-5 split for the final fold. Final model was chosen based on best model performance on 4-fold validation dataset. All images were color normalized for consistency, were randomly augmented to prevent overfitting on the training dataset, and an inverse proportional weighted sampler was used to prevent overlearning on the longest phase or step. Accuracy, precision (aka positive predictive value), recall (aka sensitivity), and F1 score (ie, the harmonic mean of precision and recall) are reported, using the following formulae^14^:

Accuracy=(true positive+true negative)/(true positive+true negative+false positive+false negative)

Precision=true positive/(true positive+false positive)

Recall=true positive/(true positive+false negative)

F1 score=2×(precision×recall)/(precision+recall)

These were calculated by comparing model predictions with the ground-truth labels. F1 score was our primary evaluation metric, as commonly used in step recognition because it limits both small precision and small recall.^31^

**FIGURE 1.**
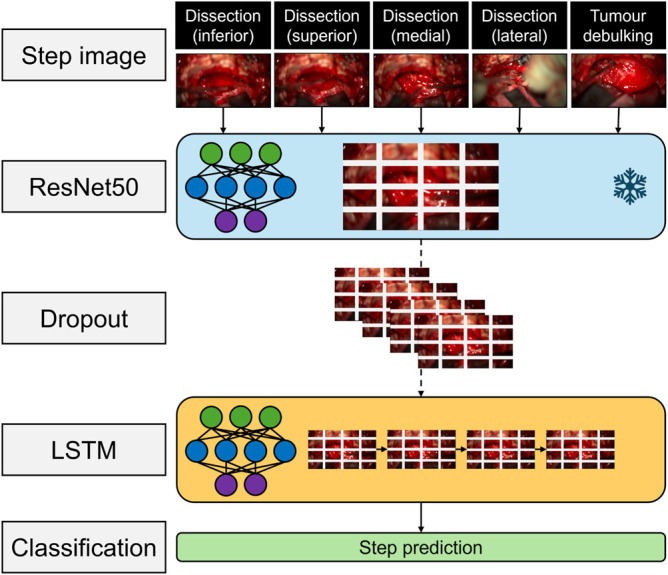
Overview of video processing architecture incorporating ResNet50 and LSTM layers. LSTM, long–short-term memory.

All 3 models used the same 2-stage architecture (Figure 1): (1) A sequence of consecutive images were sent through a convolution neural network (CNN) to extract the spatial features; (2) these features were fed into a long–short-term memory network (LSTM), a type of recurrent neural network (RNN), to extract temporal features; and these features were sent to a final classification layer which predicted the final phase or step. The input is passed as a 224 × 224 image into the ResNet50 backbone after random resizing and cropping. The ResNet50 processes the input, applying multiple convolution layers, batch normalization, and pooling. Before reaching the final fully connected layer, a global average pooling layer reduces the spatial dimensions of the feature map into a single vector of size 2048. This single vector is passed through to a fully connected layer, which reduces the dimensions to 512 and applies ReLU activation to introduce nonlinearity and 50% dropout for regularization. This output incorporates spatial information and then feeds into a LSTM structure, which models the temporal dependencies. Hyperparameter tuning was performed to identify optimal parameters in the training parameters, such as learning rate, momentum, weight decay, and to identify optimal parameters in the LSTM layer structure, such as hidden size (which determines the dimensionality of the hidden and cell states) and batch size. Those parameters that led to the highest training accuracy and validation F1 metric were selected. Weights from the CNN (ResNet50) were first trained and then frozen during LSTM training. A 50% dropout was used to prevent overfitting on the training dataset. The optimizer used was Stochastic Gradient Descent with an initial learning rate of 0.01, a momentum of 0.9, and a decay down to a minimum of 0.00001. The loss function was cross-entropy, run with a batch size of 30 for 50 epochs, with an early stopping possible after 30 epochs.

## RESULTS

### Baseline Data

Twenty-one operative videos of retrosigmoid resection to VS were collected. Baseline demographic data are provided in Table 3. Eight patients had a maximal tumor diameter of >30 mm, 12 patients 20-30 mm, and 1 patient <10 mm (**Supplemental Digital Content 3** [http://links.lww.com/NEU/E754]).

**TABLE 3.** Baseline Demographic and Surgical Data

Number of patients included	N = 21
Age (median + IQR)	59 (49-64)
Sex	
Male	13 (62%)
Female	8 (38%)
Tumor laterality	
Left	10 (48%)
Right	11 (52%)
Prior treatment	
No prior treatment	18/21
Previous Gamma Knife	2/21
Previous resection	1/21
Preoperative tumor size (median + IQR)	10.9 cm^3^ (9.0-18.9)

### Video Characteristics

The median operative video time was 5 hours 18 minutes (IQR 3 hours 21 minutes-6 hours 1 minute). All operative videos were complete, including all 3 predefined phases (Approach and Exposure, Tumor Excision, and Closure). Phase transitions were linear for all videos (ie, all videos went sequentially through each phase, with no instances of returning to the prior phase). The median duration was 28 minutes (IQR 8 minutes-31 minutes) for the “Approach and Exposure” phase, 4 hours 23 minutes (IQR 2 hours 23-4 hours 54) for the “Tumor Excision” phase, and was 17 minutes (IQR 14 minutes-27 minutes) for the “Closure” phase.

**FIGURE 2.**
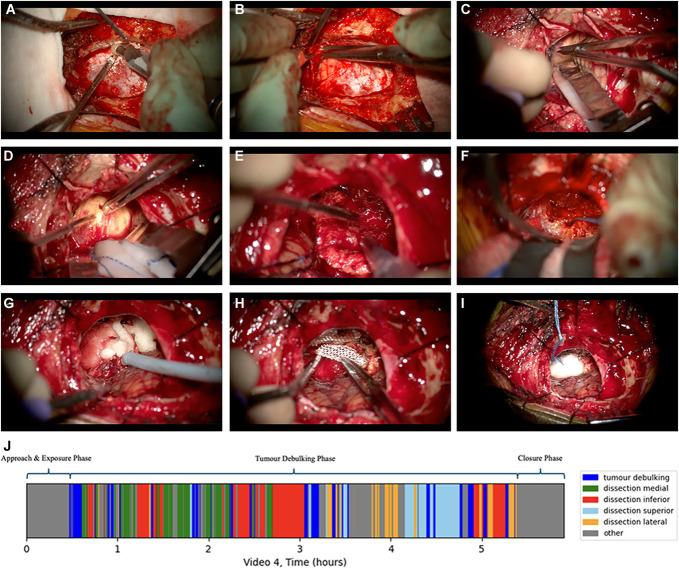
Typical operative images for each surgical phase. **A**-**C**, in the “Approach and Exposure” phase, we see several steps, including retrosigmoid craniotomy using a (**A**) diamond burr, (**B**) durotomy, and (**C**) sharp arachnoid dissection to open cisterna magna for a left-sided vestibular schwannoma. Retroauricular skin incision, retraction, and much of the initial craniotomy was not performed microscopically and was therefore absent from operative videos. **D**-**F**, the “Tumor Excision” phase was comprised several steps, including “Tumor Debulking Step,” typically involving excision of tumor using (**D**) microscissors or (**E**) Cavitron Ultrasonic Surgical Aspirator and (**F**) superior, inferior, medial, and lateral dissection, typically performed using Rhoton microdissectors. **G**-**I**, the “Closure” phase involved hemostasis using hemostatic adjuncts such as (**G**) Floseal® (Baxter), (**H**) Surgicel™ (Ethicon, Johnson & Johnson), and (**I**) cotton wool balls. Dural closure and skin closure was typically not performed microscopically and is therefore absent from the operative videos. Images displayed are from several different operative videos. All feature left-sided approaches. **J** shows a typical time plot showcasing the duration of each phase and the interchange between steps within the “Tumor Excision” phase.

Within the “Tumor Excision” phase, there were 5 labelled steps—tumor debulking, and superior, inferior, medial, and lateral dissection. Table 4 presents the number of discrete episodes of each step, and the median time spent on each step. There was a median of 78 step transitions during the tumor excision phase. Figure 2 shows typical operative images for each surgical phase, along with a typical distribution of steps.

**TABLE 4.** “Tumor Excision Phase” Operative Step Video Characteristics Ordered by Frequency

Operative step	Total no. of frames^a^	No. of discrete episodes per video (median + IQR)	Duration of each discrete episode (min) (median + IQR)	Average total duration per operation (min)	Average percentage of tumor excision phase (%)
Medial dissection	77 001	21 (12-28)	1.8 (0.8-3.6)	61	33
Inferior dissection	54 310	16 (13-21)	1.6 (0.6-3.6)	43	23
Tumor debulking	53 325	21 (16-30)	1.4 (0.8-2.4)	42	23
Lateral dissection	28 427	10 (4-13)	1.1 (0.5-2.8)	23	12
Superior dissection	23 632	10 (7-12)	1.4 (0.5-3.2)	19	10

a Frames extracted at 1 frame per second.

### Model Performance: Phase Prediction

**FIGURE 3.**
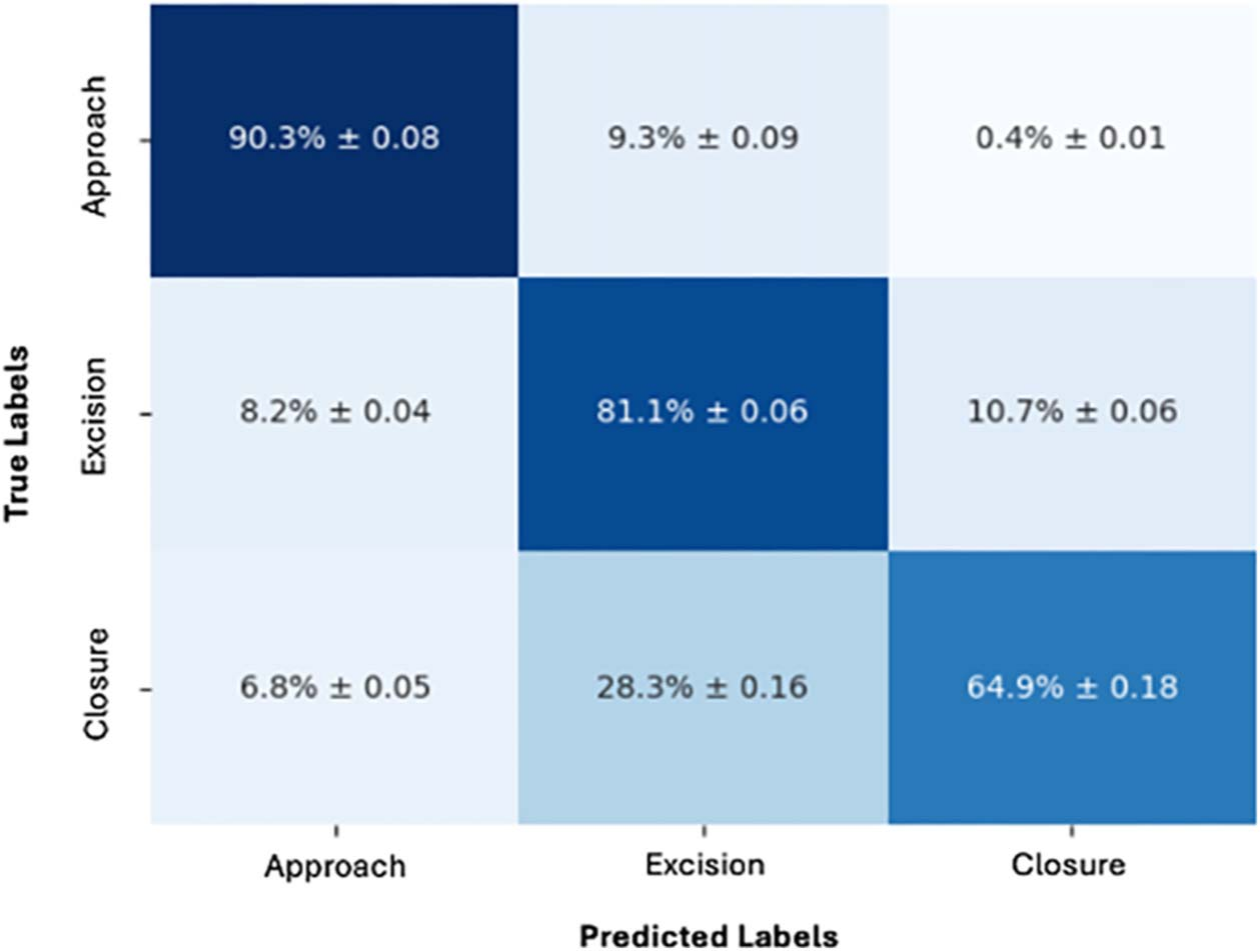
Confusion matrix showing accuracy and SDs for operative phase prediction.

For phase prediction, iteration of our model, including modifying our frame rate to coarser 1 frame per 25 seconds, resulted in recall values of 0.90 (±0.08) for the approach phase, 0.81 (±0.06) for the tumor excision phase, and 0.65 (±0.18) for the closure phase (Figure 3), giving an overall weighted average F1 score of 0.83 (±0.02), precision of 0.88 (±0.01), and recall of 0.81 (±0.03, Table 5).

**TABLE 5.** Summary of Performance Metrics for Workflow Classification in Retrosigmoid Vestibular Schwannoma Surgery

Workflow component	Accuracy	Precision	Recall	F1 score
Phase	0.81 (±0.03)	0.88 (±0.01)	0.81 (±0.03)	0.83 (±0.02)
Dichotomized steps^a^	0.86 (±0.02)	0.86 (±0.02)	0.86 (±0.02)	0.86 (±0.02)
Steps	0.59 (±0.08)	0.64 (±0.09)	0.59 (±0.08)	0.58 (±0.07)

a Binary review of the model in delineating between tumor debulking steps and all combined dissection steps.

### Model Performance: Dichotomized Step Prediction: Combined Dissection vs Debulking

Next, we binarily reviewed the performance of the model in delineating between tumor debulking steps and all combined dissection steps. Our iterated LSTM model showed a recall of 92.8% (±0.03) for dissection and 63.2% (±0.12) for tumor debulking, resulting in an overall accuracy of 0.86 (±0.02), a combined weight average precision of 0.86 (±0.02), recall of 0.86 (±0.02), and weighted average F1 of 0.86 (±0.02).

### Model Performance: Individual Step Prediction

**FIGURE 4.**
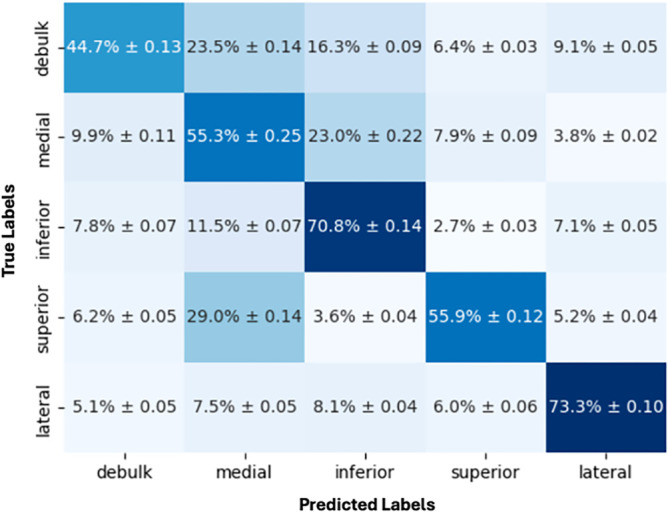
Confusion matrix showing accuracy and SDs for operative step prediction.

Finally, our team reviewed the ML model's performance on individual steps (ie, tumor debulking and superior, inferior, medial, and lateral dissection). For step prediction (within the tumor excision phase), the model achieved a weighted average accuracy of 0.59 (±0.08), precision of 0.64 (±0.09), recall of 0.59 (±0.08), and weighted average F1 score of 0.58 (±0.07) (Table 5). Lateral dissection was the most accurately predicted step (73.3% accuracy), followed by inferior dissection (70.8%), superior dissection (55.9%), medial dissection (55.3%), and tumor debulking (44.7%) (Figure 4).

## DISCUSSION

### Principal Findings

This study describes a CNN-RNN deep learning architecture for predicting the operative workflow of VS resection. This work addresses unique computer vision challenges: first, the analysis of extensive datasets (median operative video length 5 hours and 18 minutes), in contrast to previous clinical applications of computer vision unanimously conducted on shorter duration procedures; and second, the navigation of surgeries lacking a linear progression of steps, exemplified by the unpredictable interchange between dissection and debulking steps during tumor excision.

Our model exhibited notable accuracy in predicting operative phases (F1 0.83). This achievement is significant, given the substantial variation in duration of each phase, with both the approach and closure phases comprising <10% of the total operative time individually. The greater accuracy observed in predicting the approach phase aligns with its distinctive visual characteristics, often involving discernible features such as different textures, including bone and visible cerebellum. Conversely, the lower accuracy in predicting the closure phase may be attributed to the visual similarities shared with the tumor excision phase.

Our model demonstrated moderate accuracy when evaluating dichotomized steps (F1 0.86). However, our model was less accurate in predicting individual steps (ie, superior, inferior, medial, lateral dissection and debulking; F1 0.58). While lateral and inferior dissection yielded accuracies of >70%, perhaps explained by the distinct bony anatomy associated with these regions, overall accuracy was poor. The decision to aggregate granular procedural steps into broad groups to enhance ML analysis has been trialed in several other publications.^32,33^ It is notable that our model used an LSTM architecture for this, demonstrating the importance of context-specific predictions in data-heavy operative video analysis. Moreover, our dichotomized step prediction methodology solely relied on the segmentation and classification of surgical steps, without leveraging additional labels such as anatomy, instrumentation, or gesture-based cues, as demonstrated in prior studies.^34^ Gradient-weighted Class Activation Maps of step predictions are provided in **Supplemental Digital Content 4** (http://links.lww.com/NEU/E755), with further analysis. Further gains in step prediction accuracy may be realized through the addition of instrument labelling or gesture/action labelling.^24,34^

### Making the Translational Leap: Real-World Applications of Automated Workflow Analysis

CV holds significant potential in surgery, but despite much promising research, there has yet to be adoption in the modern operating theatre. Key areas for implementation of CV-based workflow technology are in training, decision support, outcome prediction, and operational efficiencies. In this study, we review how this technology affects these areas, along with real-world examples of clinical translation.

Surgical excision of VS is a high-risk, complex operation that is typically not performed until late in neurosurgical training. Limited exposure to complex cases such as VS resection have clear implications for patient safety, particularly given evidence supporting the presence of a prolonged learning curve for VS, evidenced by a strong association with improved outcomes in high volume centers.^35,36^ Automated operative video analysis has the potential to address these concerns through improved training.^9,22,24^ First, automated indexing of cases can create a real-time bank of operative videos for teaching and flag challenging cases for review,^7^ enabling trainees to receive feedback on surgical technique from trainers in a more controlled, reflective environment.^22^ Second, and in contrast to the traditional approach of using operative videos to analyze *surgeon*-related outcomes (eg, technique, skill), automated operative video analysis may be linked to *patient* outcomes. Surgeons and patients alike may benefit from retrospective review of surgical video in cases with poor outcomes, breaking the delayed feedback loop that limits educational gains. Finally, automated performance assessment through operative video analysis may facilitate benchmarking of surgical skill acquisition. Overall, through automated video analysis, trainees may be afforded serial monitoring of performance, with enhanced, retrospective, direct linkage to patient outcomes, facilitating learning and enabling a more concise estimate of the procedure specific gradient of their learning curve.^22,37^

While nascent, several real-world examples have appeared in the literature wherein these training applications are examined. Cizmic et al^38^ recently published the results of a randomized control trial in which trainees performing a laparoscopic cholecystectomy were allocated to a structured video debrief featuring video annotation or to a control group. Participants in the video-training group were found to have overall greater performance in both general and task-specific measures of surgical performance. Khan et al^39^ developed an AI-assisted video-based surgical coaching program for trainee neurosurgeons performing pituitary surgery and demonstrated both improved objective surgical performance and a reduction in postoperative complications such as postoperative anterior pituitary hormone deficit. Other use-cases, including large scale multicenter studies, have been reported.^40^

Beyond training, decision support represents another pivotal application of CV in surgical video analysis.^9^ For trainees, we envisage this to involve displaying the surgical workflow alongside guidance for the next operative step, whereas for senior surgeons, step and phase metrics may help identify outlier cases. Outcome prediction represents another promising application of CV, with automated, real-time analysis of phase and step durations or transitions potentially correlating with clinical outcomes. For example, extended tumor debulking times may be associated with complications such as cerebrospinal fluid leaks. Such applications are being explored within our own group—Khan et al^41^ demonstrated that video analysis of surgical technique and phase/step duration were associated with superior surgical outcomes in endoscopic pituitary surgery.

Machine-learning workflow analysis may also improve the efficiency of surgical systems. Automated review of the operative workflow may benefit OR staff by orientating the team to the relevant stage of an operation, enabling enhanced preparedness during critical steps of procedures.^14^ Moreover, automated linkage of the operative workflow to a hospital's electronic surgical planning system may enhance operating room turnover—for example, historical and real-time data from automated workflow systems can help predict surgery durations more accurately, improving overall scheduling efficiency and reducing OR idle time.^42,43^ Similar analysis may help to streamline resource management, as accurately predicting the required duration and materials for a surgery, hospitals can better allocate resources such staff time, OR scheduling, and consumables. So-called “Smart” operating rooms are increasingly becoming a reality.^44^

Translating these applications into clinical practice is essential, and lessons can be drawn from advancements in diagnostic imaging computer vision platforms. A notable example is the ENDO-AID Computer-Assisted Detection/Diagnosis (CADe) platform for detecting potentially neoplastic colorectal lesions, now commercially available. Following promising retrospective preclinical studies (IDEAL Stage 0), which demonstrated a significant improvement in polyp detection rates (64.7% vs 46.0%),^45^ external validation^46^ and IDEAL Stage 2-3 evaluations, including a randomized controlled trial, confirmed its efficacy in increasing adenoma detection rates.^47^ Successful clinical translation of surgical video analysis technologies similarly requires stepwise evaluation through validated frameworks such as the IDEAL framework, beginning with early comparative studies to demonstrate the technology's benefits and progressing to its integration into live clinical settings. Indeed, our own group have followed this evaluative pathway; Khan et al demonstrated that AI-assisted anatomy recognition during endoscopic pituitary surgery significantly benefits surgeons of all levels^48^; while improvement in cerebral aneurysm detection has been demonstrated among the wider surgical team with AI-assistance during aneurysm clipping operations (in press). Crucially, translational research, particularly in early phases, must place a heavy focus on the implementation of technologies, including feasibility, acceptability, and impact on surgical performance.^39^

### Comparison to Current Literature

Within the subfield of automated operative workflow analysis, we can draw comparison across 3 areas: dataset, architecture, and results. Our dataset bears several differences compared with the literature, including operative video duration (our median operative video length is 318 minutes, significantly longer than in the existing literature^14,49-51^), frequent step transition, imaging modality (microsurgery vs endoscopic), and its application to lateral skull base surgery, the first of its kind in the literature. This introduces several additional computer vision challenges, such as computational feasibility, temporal consistency, and generalizability of models across different types of surgery. Our use of CNN-RNN LSTM architecture has been used for automated operative workflow prediction in the past.^14,24,52^ Regarding results, although achieving a phase prediction accuracy of 0.81 and a dichotomized step prediction accuracy of 0.86 aligns with findings in the published literature,^14,33,50^ it is crucial to acknowledge the lower accuracy in predicting individual steps. Iterative enhancements in our model's performance could be achieved through advanced feature engineering and leveraging transfer learning from other lateral skull base datasets.

### Limitations

Despite each surgical video containing approximately 5 hours of video data, our dataset is small, and all derived from a single institution, which limits model training and increases risk of overfitting. Owing to the limited dataset, we were constrained to evaluate only a select number of surgical steps, potentially overlooking the nuances of the entire surgical workflow. Furthermore, the model's effectiveness may be compromised by its inability to capture the entirety of the surgical procedure (ie, initial stages of approach and final stages of closure not captured in operative video). This study describes the development and training of a ML model, and other than cross-fold validation, does not feature internal/external validation, inherently limiting the wider applicability of our platform. Further robust validation is critical before clinical translation.

In future, we aim to establish a collaborative network for video sharing comprising surgical institutions worldwide. This network will enable the curation of an extensive operative video database and facilitate external prospective validation of our model. Robust, stepwise evaluation in the preclinical stage (IDEAL Stage 0) will facilitate progression to first-in-human applications (IDEAL Stage 1), such as real-time workflow analysis.

## CONCLUSION

This study demonstrates the ability of a CNN-RNN deep learning model to predict the surgical phases of retrosigmoid VS resection with high accuracy. In addition, our model showed moderate-to-high accuracy in discerning intraphase steps of dissection vs tumor debulking. In doing so, this study acts as a showcase for ML-based operative workflow recognition in low-volume, high complexity operative procedures that involve prolonged duration and data-heavy operative video datasets. Automated ML-based recognition of the operative workflow holds significant potential in numerous applications, including training, theatre efficiency, and intraoperative guidance. Future work should focus on leveraging this technology to realize these applications, while incorporating automated workflow technology into live operating room settings.

## Supplementary Material

## SUPPLEMENTAL DIGITAL CONTENT

**Supplemental Digital Content 1**. Standard operating procedure for retrosigmoid excision of vestibular schwannoma. **Supplemental Digital Content 2**. Retrosigmoid operative workflow. Tables reproduced from Layard Horsfall H, Khan DZ, Collins J, et al. Generating operative workflows for vestibular schwannoma resection: a two-stage Delphi's consensus in collaboration with the British Skull Base Society. Part 1: The Retrosigmoid Approach. *Journal of Neurological Surgery. Part B, Skull Base*, 84(5), 423-432. https://doi.org/10.1055/a-1886-5500. Subheadings: Phase 1: Approach and Exposure. Phase 2: Tumor debulking and excision. Phase 3: Closure. **Supplemental Digital Content 3**. Preoperative tumor measurements. **Supplemental Digital Content 4**. Gradient-weighted class activation maps.
