# Supplementary material for: Automated Operative Phase and Step Recognition in Vestibular Schwannoma Surgery: Development and Preclinical Evaluation of a Deep Learning Neural Network (IDEAL Stage 0)
Source: Neurosurgery. 2025 Apr 30;98(4):799–809. doi: 10.1227/neu.0000000000003466 (PMC12962350; doi:10.1227/neu.0000000000003466)
Supplement: Supplementary file 1 [file neu-98-799-s001.docx]

# Supplemental Digital Content 1: Standard Operating Procedure for Retrosigmoid Excision of Vestibular Schwannoma

Decision to operate is made in conjunction with the patient and the multidisciplinary team (MDT) consisting of skull base neurosurgeons, otorhinolaryngologists, neuroradiologists, and clinical nurse specialists. Prior to operation, all patients were seen in an anaesthetic pre-assessment clinic. All patients underwent general anaesthesia, and were positioned in a park bench position, before undergoing skin antisepsis and draping. Neuromonitoring was used throughout to monitor the trigeminal, facial, and lower cranial nerves. A retroauricular curvilinear incision is made down to bone, followed by a retrosigmoid craniectomy using a diamond cutting burr (Bien Air Surgery, Switzerland). The bone dust is stored for later use. Typically, at this stage, the operating microscope is introduced and operative video recording begins. The transverse-sigmoid junction is identified, before a cruciate durotomy is performed. The cerebellum is gently retracted, and the cisterna magna is opened using a sharp hook or micro-scissors, to enable CSF egress. A fixed retractor is used to retract the cerebellum medially to reveal the tumour. The superior and inferior poles are dissected, and the lower cranial nerves and posterior inferior cerebellar artery (PICA) are identified, if possible prior to debulking. The tumour is then debulked internally, using a combination of instruments including Spetzler-Malis bipolar forceps, microscissors, Cavitron Ultrasonic Surgical Aspirator (CUSA), and Rhoton microdissectors. The tumour rind is progressively rolled from side to side with gentle dissection of each pole (superior, inferior, medial, lateral). If necessary, the internal acoustic canal is drilled to enable visualisation of the facial nerve and tumour origin. Facial nerve stimulation is used throughout. Depending on the surgical goal, a subtotal, gross total, near total, or total resection may be achieved. During the closure phase, meticulous haemostasis is achieved using a variety of methods, including use of bipolar and haemostatic adjuncts (including but not limited to Floseal® (Baxter, UK) (G), Surgicel™ (Ethicon, Johnson & Johnson, USA) (H), and cotton wool balls). At this point the operating microscope is removed from use, ending the operative video. The dural leaflets are sutured together with 3-0 vicryl and Duragen® (Integra LifeSciences, USA) is laid over the dura. Bone dust is placed over the craniectomy site and is layered with Tisseel® (Baxter, UK). Vicryl (2-0) sutures are used close the muscle, galea and subcutaneous layers. Surgical clips (Ethicon Endo-Surgery, USA) are used to close the skin. A head bandage is placed and patients are extubated and taken to surgical recovery, before being taken to the intensive care unit (ICU) for routine monitoring. Patients are typically discharged from ICU to the ward the next day. During the post-operative period, patients undergo assessment by occupational therapists, physiotherapists, speech and language therapists, and the neurosurgical team, prior to discharge.
