# Supplementary material for: Automated Operative Phase and Step Recognition in Vestibular Schwannoma Surgery: Development and Preclinical Evaluation of a Deep Learning Neural Network (IDEAL Stage 0)
Source: Neurosurgery. 2025 Apr 30;98(4):799–809. doi: 10.1227/neu.0000000000003466 (PMC12962350; doi:10.1227/neu.0000000000003466)
Supplement: Supplementary file 2 [file neu-98-799-s002.docx]

# Supplemental Digital Content 2: Retrosigmoid Operative Workflow

**Phase 1: Approach and Exposure**

| **#** | **Steps** | **Instruments** | **Technique error** | **Event error** |
| --- | --- | --- | --- | --- |
| 1 | Retroauricular incision to bone | Scalpel, monopolar, retractors | - Vertebral artery injury | - Vertebral artery bleeding or infarct |
| 2 | Haemostasis | Monopolar, bipolar, suction, bone wax |  |  |
| 3 | +/- Retrosigmoid craniectomy +/- collection of bone dust | Cutting burr, Kerrison punch, periosteal elevator, bone wax | - Dural sinus injury - Opening of mastoid air cells without repair | - Haemorrhage - Air embolism - Sinus thrombosis - CSF rhinorrhoea |
| 4 | +/- Retrosigmoid craniotomy | Perforator, Penfield dissector, craniotome, bone wax | - Dural sinus injury - Opening of mastoid air cells without repair | - Haemorrhage - Air embolism - Sinus thrombosis - CSF rhinorrhoea |
| 5 | Seal mastoid air cells | Bone wax | - Failure to seal mastoid air cells | - CSF rhinorrhoea |
| 6 | Durotomy | Scalpel, blunt hook, cottonoid patties, dural scissors | - Dural sinus injury | - Haemorrhage - Air embolism - Sinus thrombosis |
| 7 | Suture to dural edges | Suture | - Dural sinus injury | - Haemorrhage - Air embolism - Sinus thrombosis |
| 8 | Cisterna magna opening | Microscope, brain retractor, scalpel, sharp hook, cottonoid patties | - Failure to open cisterna magna - Insufficient CSF egress - Excessive retraction | - Cerebellar swelling and retraction injury - Haemorrhage |
| 9 | Approach to cerebellopontine angle & retraction of cerebellum | Microscope, microdissector, microscissors, suction, retractors, linteens, cottonoid patties | - Stretching of cranial nerves (CN) - Excessive retraction | - CN VII, XI, X palsy - Superior petrosal vein injury - Tearing of bridging veins and haemorrhage - Cerebellar swelling and retraction injury |
| 10 | Dissection of arachnoid plane from tumour capsule | Microscope, bipolar, suction, microdissector, microscissors, cottonoid patties | - Loss of arachnoid plane or entry into incorrect plane - Excessive traction on capsule | - Haemorrhage - CN injury |

**Retrosigmoid Operative Workflow**

**Phase 2: Tumour debulking and excision**

| **#** | **Steps** | **Instruments** | **Technique error** | **Event error** |
| --- | --- | --- | --- | --- |
| 1 | Posterior aspect of tumour stimulated for facial nerve | Microscope, facial nerve stimulator | - Failure to identify CN VII | - CN VII palsy |
| 2 | Tumour capsule opened and primary debulking | Microscope, bipolar, suction, microdissector, cottonoid patties, ultrasonic aspirator, tumour holding forceps | - Incomplete haemostasis - CN injury | - Haemorrhage - CN palsy |
| 3 | +/- Tumour biopsy | Tumour holding forceps |  |  |
| 4 | Inferior pole resection and separation from lower cranial nerves and vessels | Microscope, bipolar, suction, microdissector, microscissors, cottonoid patties, ultrasonic aspirator, tumour holding forceps | - Injury to CN IX, X, XI - Injury to vessels: AICA, PICA | - CN IX, X, XI palsy - Haemorrhage - Infarct |
| 5 | Identification of CN VIII at brainstem and dissection of arachnoid medially | Microscope, bipolar, suction, microdissector, microscissors, cottonoid patties, knife | - Incorrect arachnoid plane - Perforating vessel injury - Injury to CN VII or VIII | - Brainstem, peduncle infarct - CN VII palsy - Hearing loss if preservation intended |
| 6 | +/- identification of dorsal cochlear nucleus for DNAP electrode if considering cochlear preservation | DNAP electrode |  |  |
| 7 | Identification of the root entry of CN VII which lies ventral and inferior to root entry of CN VIII | Microscope, bipolar, suction, microdissector, microscissors, cottonoid patties | - Vessel injury - Injury to CN VII | - Haemorrhage or infarct - CN VII palsy |
| 8 | +/- FREMAP electrode | FREMAP electrode |  |  |
| 9 | Superior pole resection | Microscope, bipolar, suction, microdissector, microscissors, cottonoid patties, ultrasonic aspirator, tumour holding forceps | - Injury to CN V or VII - Injury to petrosal vein or SCA | - CN V, VII palsy - Haemorrhage - SCA infarct |
| 10 | Identification and protection of petrosal vein +/- coagulation and division of petrosal vein only if absolutely necessary | Microscope, bipolar, suction, microdissector, microscissors, Cottonoid patties, scalpel | - Traction on petrosal vein - Injury to SCA - Sinus injury | - Venous infarct or haematoma - Air embolism - Sinus thrombosis |
| 11 | Dissection of tumour capsule from CN V | Microscope, bipolar, suction, microdissector, microscissors, cottonoid patties | - Injury to CN IV or V - Injury to SCA | - CN IV or V palsy - SCA infarct |
| 12 | Medial to lateral dissection and rolling of the tumour from cerebellar peduncle and brain stem | Microscope, bipolar, suction, microdissector, microscissors, cottonoid patties, tumour holding forceps, ultrasonic aspirator | - CN VII injury at root entry zone - Injury to perforating vessels | - CN VII palsy - Peduncle or brainstem infarct |
| 13 | Drilling of internal auditory canal | Drill, irrigation, (+/- cutting, +/- diamond burr), curette, bone wax | - Air cell opening without repair - Opening of the labyrinthe or endolymphatic duct - Jugular bulb injury | - Hearing loss - CSF leak - Haemorrhage - Air embolism |
| 14 | Incise dura of IAM and reflect away from tumor | Drill, irrigation, (+/- cutting, +/- diamond burr), curette, bone wax, facial nerve stimulator | - CN injury - Vessel injury | - Haemorrhage - CN palsy |
| 15 | Locate fundus of IAM and dissect superior vestibular nerve as laterally as possible | Microscope, bipolar, suction, microdissector, microscissors, cottonoid patties, knife |  |  |
| 16 | +/- Sacrifice of cochlear nerve in large tumours | Microscope, bipolar, suction, microdissector, microscissors, knife, blunt hook, facial nerve stimulator | - Failure to identify CN VII in distal canal as distinct from tumour and other CN - Injury to cochlear nerve in attempted hearing preservation surgery | - CN VII palsy - Hearing loss |
| 17 | Continue dissection with lateral to medial dissection to the porous | Microscope, bipolar, suction, microdissector, microscissors, cottonoid patties, knife | - Failure to keep CN VII visualised at all times - Failure to maintain plane between tumour and CN VII | - CN VII palsy |
| 18 | Resection of tumour in the CPA until lateral-medial and medial-lateral dissections to join together | Microscope, bipolar, suction, microdissector, microscissors, cottonoid patties, ultrasonic aspirator, tumour holding forceps |  |  |
| 19 | Removal of tumour after stepwise rolling and debulking of tumour as above | Microscope, bipolar, suction, microdissector, microscissors, cottonoid patties, ultrasonic aspirator, tumour holding forceps | - CN injury - Vessel injury - Brainstem or peduncle injury | - Haemorrhage - CN injury - Brainstem or peduncle oedema or infarct |
| 20 | +/- in circumstance when facial nerve is not preserved, perform facial nerve graft (proximal and distal stump anastomosis using nerve +/- sural or greater auricular nerve) | Scalpel, monopolar, retractor, microscope, suture | - Incomplete anastomosis - CN injury | - CN VII palsy |

*NB: We appreciate the exact order of the following steps will be surgeon and tumour characteristic dependent.*

*Abbreviations: AICA, anterior inferior cerebellar artery; CN, cranial nerve; CPA, cerebellopontine angle; CSF, cerebrospinal fluid; DNAP, dorsal*

*cochlear nucleus action potential; FREMAP, facial nerve root exit zone–elicited compound muscle action potential; IAM, internal auditory meatus;*

*IQR, interquartile range; PICA, posterior inferior cerebellar artery; SCA, superior cerebellar artery.*

**Retrosigmoid Operative Workflow**

**Phase 3: Closure**

| **#** | **Steps** | **Instruments** | **Technique error** | **Event error** |
| --- | --- | --- | --- | --- |
| 1 | CN VII stimulation to confirm response at low level (0.05mA) | Facial nerve stimulator | - No stimulation | - CN VII palsy |
| 2 | Haemostasis | Bipolar, fibrin sealant, oxidised cellulose matrix, cottonoid patties | - Incomplete haemostasis | - Haematoma |
| 3 | Seal mastoid air cells | Bone wax, fibrin glue | - Failure to seal mastoid air cells | - CSF leak |
| 4 | Resection cavity inspection |  | - Failure to identify residual tumour | - Recurrence or incomplete tumour resection |
| 5 | Dural repair | Suture, +/- synthetic dural substitute, +/- dural sealant glue | - Incomplete closure | - CSF leak - Pseudomeningocoele |
| 6 | +/- Replacement of bone flap | Bone flap, miniplates, screws, +/- bone substitute | - Incomplete closure | - CSF leak - Pseudomeningocoele |
| 7 | +/- Replacement of bone dust or bone cement | Bone dust or bone cement | - Incomplete closure | - CSF leak |
| 8 | Closure of muscle layer and fascia | Suture | - Incomplete closure | - CSF leak - Infection |
| 9 | Skin closure | Suture, clips | - Poor opposition of skin edges | - CSF leak - Wound infection |
