# Supplementary material for: Automated Operative Phase and Step Recognition in Vestibular Schwannoma Surgery: Development and Preclinical Evaluation of a Deep Learning Neural Network (IDEAL Stage 0)
Source: Neurosurgery. 2025 Apr 30;98(4):799–809. doi: 10.1227/neu.0000000000003466 (PMC12962350; doi:10.1227/neu.0000000000003466)
Supplement: Supplementary file 3 [file neu-98-799-s003.docx]

# Supplemental Digital Content 3: Pre-Operative Tumour Measurements

| Table in Appendix 3: Pre-Operative Tumour Measurements | | | | | | | | |
| --- | --- | --- | --- | --- | --- | --- | --- | --- |
| Patient Number | Age | Sex | Tumour Laterality | Koos Grade | Pre-operative Tumour size | | | Pre-operative volume* (cm3) |
|  |  |  |  |  | **AP (mm)** | **TR (mm)** | **CC (mm)** |  |
| ** Tumour volumes calculated using volume of ellipsoid formula - (4/3*π*a*b*c, where a b and c are the AP, TR, CC radii).* | | | | | | | | |
| Patient 1 | 55 | M | Right | 4 | 39.4 | 32.6 | 33.5 | 22.53 |
| Patient 2 | 70 | F | Left | 4 | 39.24 | 23.81 | 30.98 | 15.16 |
| Patient 3 | 49 | M | Left | 4 | 35.27 | 30.65 | 22.4 | 12.68 |
| Patient 4 | 69 | M | Left | 4 | 36.09 | 30.25 | 32.61 | 18.64 |
| Patient 5 | 38 | M | Right | 4 | 36 | 33 | 31 | 19.29 |
| Patient 6 | 21 | F | Right | 2 | 8.37 | 7.04 | 6.46 | 0.199 |
| Patient 7 | 72 | M | Right | 3 | 28.23 | 24.23 | 27.59 | 9.883 |
| Patient 8 | 59 | M | Right | 4 | 25.73 | 20.17 | 22.51 | 6.118 |
| Patient 9 | 44 | F | Left | 3 | 35.25 | 25.18 | 19.26 | 8.952 |
| Patient 10 | 64 | M | Left | 3 | 24.2 | 23.38 | 21.93 | 6.498 |
| Patient 11 | 54 | M | Left | 4 | 36.43 | 31.9 | 34.81 | 21.18 |
| Patient 12 | 60 | F | Right | 4 | 31.57 | 22.78 | 25.51 | 9.607 |
| Patient 13 | 73 | F | Right | 4 | 30.52 | 35.79 | 33.04 | 18.9 |
| Patient 14 | 23 | M | Right | 4 | 34.59 | 39.3 | 45.53 | 32.41 |
| Patient 15 | 37 | F | Left | 4 | 25.51 | 27.97 | 28.13 | 10.51 |
| Patient 16 | 67 | M | Left | 4 | 48.99 | 31.06 | 38.66 | 30.81 |
| Patient 17 | 62 | M | Left | 3 | 25 | 27.5 | 31.28 | 11.26 |
| Patient 18 | 49 | F | Right | 3 | 26 | 24 | 22.8 | 7.45 |
| Patient 19 | 62 | M | Right | 4 | 26.57 | 23.6 | 25.43 | 8.35 |
| Patient 20 | 55 | F | Right | 4 | 30 | 23.97 | 24.88 | 9.369 |
| Patient 21 | 60 | M | Left | 4 | 30.24 | 27.35 | 26.4 | 11.43 |
