# Supplementary material for: Automated Operative Phase and Step Recognition in Vestibular Schwannoma Surgery: Development and Preclinical Evaluation of a Deep Learning Neural Network (IDEAL Stage 0)
Source: Neurosurgery. 2025 Apr 30;98(4):799–809. doi: 10.1227/neu.0000000000003466 (PMC12962350; doi:10.1227/neu.0000000000003466)
Supplement: Supplementary file 4 [file neu-98-799-s004.docx]

# Supplemental Digital Content 4: Gradient-Weighted Class Activation Maps

Gradient-weighted Class Activation Maps (Grad-CAM) are provided below for intra-phase steps, to provide visualisations of the regions within each frame that are most influential in the model’s classification of surgical steps. These visualisations utilise the final ResNet50 weights and allow us to highlight areas of interest, such as instruments entering the field of view or specific anatomical landmarks, that contribute significantly to the model’s decisions. This adds a layer of explainability to the model’s predictions, and can be used as a means of failure analysis in poorer performing predictions e.g. step prediction.

The CNN component of the model focuses on spatial feature extraction, identifying key elements within the video frame, while the LSTM integrates these spatial features across time to recognise temporal patterns and ensure contextual continuity. For example, when a new surgical instrument enters the field, the CNN registers this as a critical spatial feature, while the LSTM maintains memory of this event to predict the likelihood of remaining within the same phase or transitioning to the next phase. This integration of spatial and temporal information directly impacts the model’s ability to achieve accurate phase or step classification.

Step Classification Grad-CAMs

During the tumour debulking phase, our model was tasked with classifying the surgical step being performed into one of five categories (medial dissection, lateral dissection, superior dissection, inferior dissection, or tumour debulking). Additionally, the model was evaluated on a dichotomised classification task, distinguishing between dissection and debulking. The model demonstrated moderate accuracy in the dichotomised classification (F1 score: 0.86) but showed reduced performance in predicting specific steps (F1 score: 0.58 for five-class classification).

Grad-CAM maps for these step predictions are presented in Supplemental Digital Figure 1. Several observations can be drawn from these visualisations. Instrument usage emerged as a crucial determinant for step classification. For instance, tumour debulking steps typically involve the use of ultrasonic aspirators and tumour forceps (as shown in Images A and B), whereas dissection steps predominantly utilise micro-dissectors (Images C–H). Additionally, the model’s ability to differentiate between spatial components of dissection (e.g., medial, lateral, superior, or inferior tumour dissection) relied heavily on the positioning of surgical retractors and instruments.

The medial retractor, consistently placed to retract the cerebellar hemisphere medially, serves as a critical landmark for spatial orientation. In correctly predicted frames (e.g., C, D, E, F), the model focuses on both the retractor and the instruments in use, effectively aligning the instrument’s activity with the tumour's relative position. However, in misclassified frames (e.g., G and H), the retractor becomes the sole focus, and the model fails to adequately account for lateral instrument activity, leading to incorrect predictions.

The static nature of individual frames may further complicate the classification task, as the visual appearance of a single action within one step can resemble that of another. For instance, gestures and actions at a finer granularity of workflow analysis (e.g., specific surgical actions within dissection or tumour debulking) may provide better predictive power. Labelling such actions could enhance the model’s ability to differentiate steps, as the surgical actions involved in dissection versus debulking are visually distinct when considered over time.


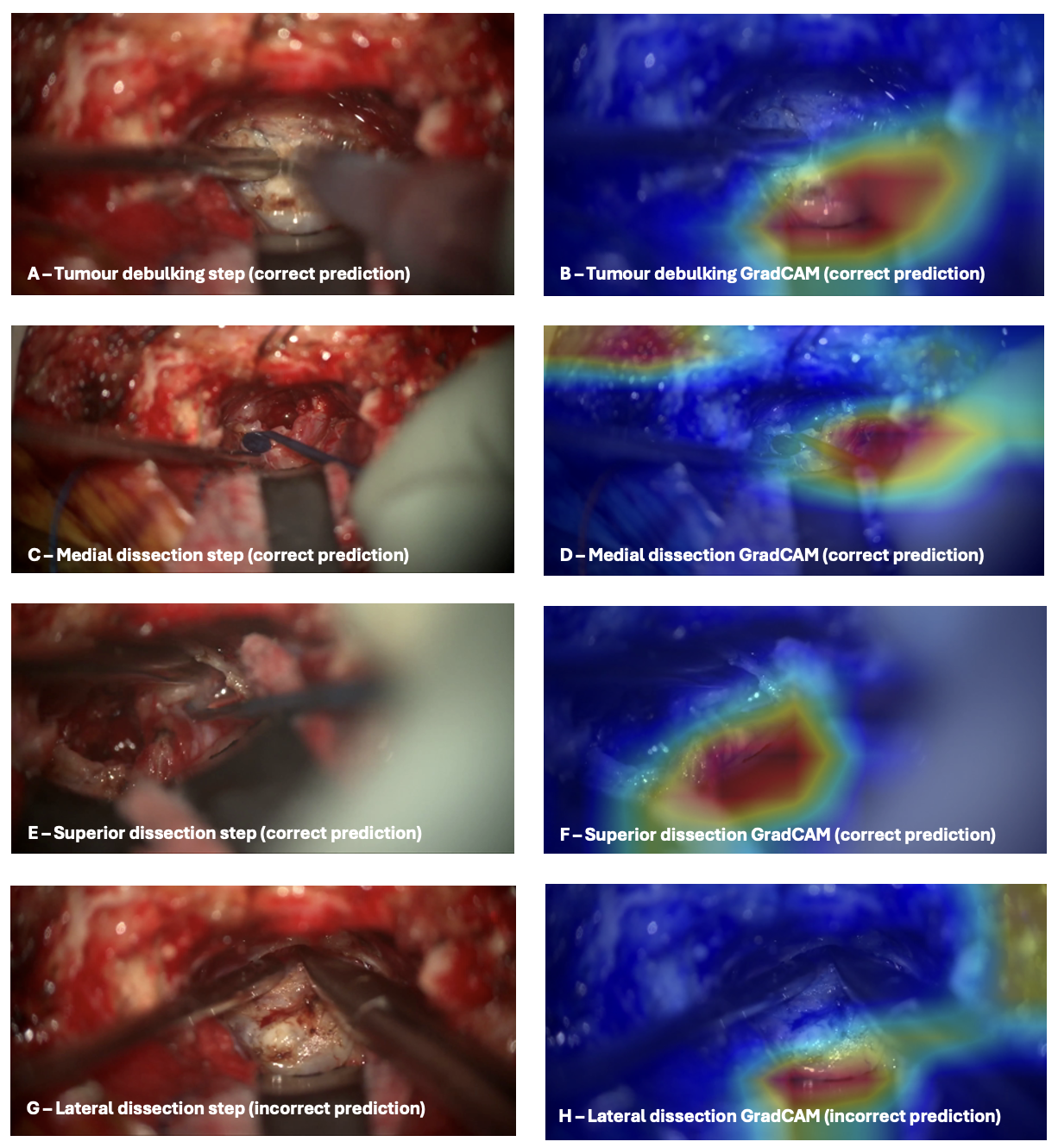


Supplemental Digital Figure 1: GradCAM maps for step prediction frames.
